# Complexes With Biologically Active Ligands. Part 1. Synthesis of Coordination Compounds of Diazoxide With Transition- and Main-Group Cations

**DOI:** 10.1155/MBD.1996.25

**Published:** 1996

**Authors:** Claudiu T. Supuran

**Affiliations:** 1 University of Florence Laboratory of Inorganic and Bioinorganic Chemistry Via Gino Capponi 7 Firenze I-50121 Italy

## Abstract

Complexes of diazoxide (3-methyl-7-chloro-1,2,4-benzothiadiazine-1,1-dioxide) - an antihypertensive and hyperglycemic pharmacological agent - with a series of transition- and main-group di-, triand tetravalent metal ions were prepared and characterized by elemental analysis, spectroscopic, thermogravimetric, magnetic and conductimetric measurements. The complexes were tested as inhibitors of the enzyme carbonic anhydrase (CA), proving modest activity towards CA II and better inhibition of CA I.


### COMPLEXES WITH BIOLOGICALLY ACTIVE LIGANDS. Part 1. SYNTHESIS OF COORDINATION COMPOUNDS OF DIAZOXIDE WITH TRANSITION- AND MAIN-GROUP CATIONS

Claudiu T. Supuran
University of Florence, Laboratory of Inorganic and Bioinorganic Chemistry,
Via Gino Capponi 7, I-50121, Firenze, Italy

**Abstract**: Complexes of diazoxide (3-methyl-7-chloro-1,2,4-benzothiadiazine-1,1-dioxide) - an anti-hypertensive and hyperglycemic pharmacological agent - with a series of transition- and main-group di-, tri- and tetravalent metal ions were prepared and characterized by elemental analysis, spectroscopic, thermogravimetric, magnetic and conductimetric measurements. The complexes were tested as inhibitors of the enzyme carbonic anhydrase (CA), proving modest activity towards CA II and better inhibition of CA I.

### Introduction

In connection with our interest to develop novel types of inhibitors of the enzyme carbonic anhydrase (CA, EC 4.2.1.1), [1,2] we reported a large number of coordination compounds of heterocyclic and aromatic sulfonamides, [2,3] containing a large range of transition and main-group metal ions. Some of these derivatives showed excellent CA inhibitory properties (for isozymes CA I and CA II, the major red cell CAs[1]) and their mechanism of action at molecular level was also rationalized. [4]

Ligands used in such studies included heterocyclic sulfonamides with well-known CA inhibitory properties, such as acetazolamide **1**;[5] methazolamide **2**; [6] ethoxzolamide **3**; [7] thienothiopyran sulfonamides **4**;[8] (all these are clinically used CA inhibitors [1,2]) as well as saccharin **5**, [9] for which CA inhibitory properties were discovered recently.[10]

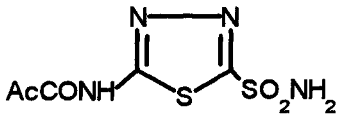

**1**

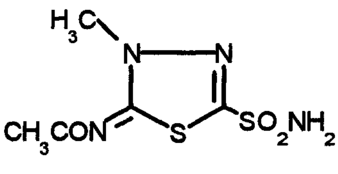

**2**

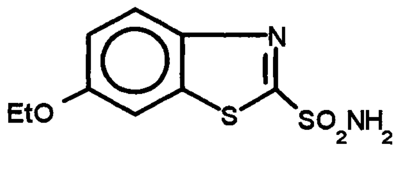

**3**

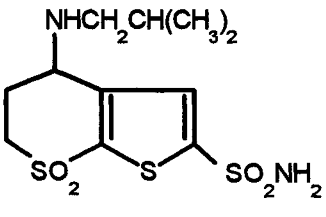

**4**

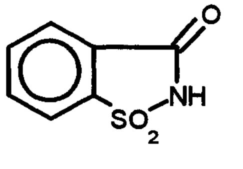

**5**

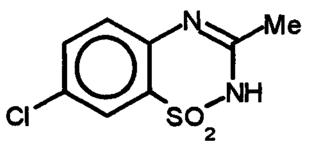

**6: HDZO**

Acetazolamide was the first non-mercurial diuretic in clinical use, [11,12] and subsequently its applications were based upon its antiglaucoma,[13] antiepileptic [14] and antiulcerogenic [15] effects. Together with the related drugs methazolamide and ethoxzolamide, they were used in clinical medicine for more than 40 years. [11] Derivative **4** was recently introduced in clinical medicine as topical antiglaucoma agents, being thoroughly effective in lowering elevated eye pressure, without undesired side effects. [13]

Acetazolamide **1** also played a major role in the development of renal physiology and pharmacology, [11,12] and led to the synthesis of two classes of drugs: the benzothiadiazide ("thiazide" diuretics) and the high-ceiling diuretics.[11,16] From the first type of such compounds, chlorothiazide (6-chloro-7-sulfamoyl-1,2,4-benzothiadiazine-1,1-dioxide) is a widely used diuretic drug for treating a variety of disorders such as edema, congestive heart failure, hypertension, etc. [15,16]

Among the large number of 1,2,4-benzothiadiazine-1,1-dioxides reported,[16] 3-methyl-7-chloro-1,2,4-benzothiadiazine-1,1-dioxide **6** (diazoxide, abbreviated as HDZO in this work) proved completely different properties as compared to the structurally related diuretics, such as chlorothiazide and its congeners (which possess a sulfamoyl group in position 7 and lack the methyl from 3). Thus, diazoxide - in clinical use since the 1960-s - is a potent hypotensive drug, its mechanism of action involving the activation of ATP-sensitive potassium channels, and relaxation of vascular smooth muscles.[17] By the same mechanism of action, diazoxide inhibits insulin secretion,[18] and shows potent hyperglycemic properties,[19] which are useful in the treatment of various forms of hypoglycemia.[19]

Although, as seen from the above data, derivatives of type **1-6** possess prominent biological activities, and are valuable pharmacological agents, used clinically for a long period, excepting for sulfonamides **1-5**, recently investigated by us, [1-10] the coordination chemistry of benzothiadiazines was not studied. Thus, a program was initiated in our laboratory to investigate the coordination chemistry of such and related derivatives, as well as the biological activity of the synthesized complexes. In this paper I report the preparation, coordination behavior and biological activity data of metal complexes of diazoxide. In future reports data for complexes of related (diuretic) benzothiadiazine-1,1-dioxides will be presented.

### Materials and Methods

FTIR spectra were obtained on thin films of pure compound, with a Perkin Elmer 1600 instrument, in the range 200 - 4000 $cm^{-1}$. Electronic spectra were obtained by the diffuse reflectance technique in MgO as reference, with a Perkin Elmer Lambda 17 apparatus. Solution electronic spectra were done in ethanol or methanol with a Cary 3 instrument. Conductimetric measurements were done in DMF solutions, at 25°C (concentrations of 1 mM of complex) with a Fisher conductimeter. Magnetic susceptibility measurements were done at room temperature by Faraday's method, using $CoHg(NCS)_4$ as standard. Elemental analyses were done by combustion for C,H,N with an automated Carlo Erba analyzer, and gravimetrically for the metal ions, and were ± 0.4% of the theoretical values. Thermogravimetric measurements were done in air, at a heating rate of 10°C/min., with a Perkin Elmer 3600 thermobalance.

Diazoxide **6** used in the syntheses was from Merck. Metal salts were from Merck, Fluka or Aldrich and were used without additional purification. Bovine CA II and human CA I were from Sigma Chemical Co. Inhibitors were assayed by Maren's micromethod[20], in the conditions of the E-I (enzyme-inhibitor) technique, at 0°C in veronal buffer. $IC_{50}$ values represent the molarity of inhibitor producing a 50% decrease of CA specific activity for the $CO_2$ hydration reaction.

### Synthesis of coordination compounds 8-17

A *cold* solution of diazoxide sodium salt (NaDZO, 7) was prepared by suspending HDZO in a 2N NaOH solution, working at 0-5°C. Mention should be made that the benzothiadiazinic ring is not very stable in the presence of bases, being cleaved to orthanilamide derivatives.[16] Still, at room temperature and in 2N NaOH, diazoxide is cleaved in about 150 hours,[16] so that, presumably, no decomposition occurred during the experiments reported here, in which complexes were prepared in about 0.5 - 1 hours. The cold solution obtained above, was mixed with a methanolic-aqueous solution of metal salts ($MCl_2$ or $MCl_3$), in molar ratios of 2:1 and 3:1, respectively, and the obtained reaction mixture was stirred magnetically at room temperature for 0.5 - 1 hours. The obtained precipitates were filtered and air-dried.

**Results and Discussion**

Starting from the sodium salt of diazoxide, NaDZO 7 , prepared in situ from diazoxide and NaOH (the $pK_a$ of the $SO_2NH$ moiety of diazoxide is 8.5)[21] both transition- as well as main-group metal complexes were obtained. Generally, cations which led to strong CA inhibitors in complexes with sulfonamides **1-5**[1-10] were included in the present study, such as Cu(II); Zn(II); Hg(II); Fe(III); V(IV), etc. The synthesized compounds **8-17** and their elemental analysis data (± 0.4% of the theoretical values, for C,H,N, by combustion, and for M by gravimetry) are shown in Table I.

Table I: The prepared diazoxide complexes **8-17**, and their elemental analysis data (DZO stands for the sulfonamide deprotonated species of the ligand).

| No. | Compound | Color | Analysis (calc./found) %M[a] | %C[b] | %H[b] | %N[b] |
|---|---|---|---|---|---|---|
| **8** | $[Co(DZO)_2(OH_2)_4]$ | violet | 9.9/9.5 | 32.5/32.4 | 3.3/3.4 | 9.5/9.2 |
| **9** | $[Ni(DZO)_2(OH_2)_4]$ | smaragd | 9.9/9.5 | 32.5/32.3 | 3.4/3.3 | 9.5/9.2 |
| **10** | $[Cu(DZO)_2(OH_2)_4]$ | gray | 10.6/10.2 | 32.3/32.1 | 3.3/3.1 | 9.4/9.1 |
| **11** | $[Zn(DZO)_2(OH_2)_2]$ | white | 11.7/11.2 | 34.3/34.2 | 2.8/2.9 | 10.0/9.7 |
| **12** | $[Cd(DZO)_2(OH_2)_4]]$ | white | 17.4/17.0 | 29.8/29.7 | 3.1/3.0 | 8.7/8.5 |
| **13** | $[Hg(DZO)_2(OH_2)_4]$ | white | 27.4/26.8 | 26.2/26.2 | 2.7/2.5 | 7.6/7.6 |
| **14** | $[Pb(DZO)_2(OH_2)_4].2H_2O$ | white | 26.7/26.3 | 24.8/24.6 | 3.1/2.9 | 7.2/7.1 |
| **15** | $[Fe(DZO)_3(OH_2)_3]$ | brown | 6.9/6.6 | 36.0/36.1 | 3.0/2.8 | 10.5/10.4 |
| **16** | $[Al(DZO)_3(OH_2)_3].6H_2O$ | white | 3.0/2.5 | 32.8/32.5 | 4.1/4.0 | 9.5/9.3 |
| **17** | $[VO(DZO)_2(OH_2)_2].H_2O$ | gray | 8.7/8.5 | 33.1/32.8 | 3.1/2.9 | 9.6/9.5 |

[a]By gravimetry; [b]By combustion

The new compounds were further characterized by IR-, and electronic spectroscopy (in solution as well as by the diffuse reflectance technique); thermogravimetric (TG) analysis, magnetic and conductimetric measurements. Some of these data are shown in Tables II and III.

Table II: Spectroscopic and TG data for compounds **6-17**.

| **Comp.** | **IR. Spectra** [a] ,$cm^{-1}$ $\nu$(M-L) | $\nu(SO_2)^s$ | $\nu(SO_2)^{as}$ | **UV Spectra** [b], $\lambda_{max}$, nm (lgε) | **TG analysis**[c] T(°C) | found /calc. |
|---|---|---|---|---|---|---|
| **6** | - | 1150 | 1342 | 215 (3.94); 278 (4.08) | - | - |
| **7** | - | 1152 | 1345 | 215 (3.96); 289 (4.23) | - | - |
| **8** | 341;398 | 1135 | 1290 | 215 (3.97); 289 (4.51) | 120-170 | 12.1/12.2[d] |
| **9** | 340;398 | 1136 | 1283 | 215 (3.96); 289 (4.53) | 120-170 | 12.0/12.2[d] |
| **10** | 310;400 | 1139 | 1286 | 215 (3.97); 289 (4.61) | 120-180 | 11.8/12.1[d] |
| **11** | 348;400 | 1137 | 1280 | 215 (3.96); 289 (4.38) | 120-160 | 6.3/6.4[e] |
| **12** | 350;400 | 1139 | 1283 | 215 (3.96); 289 (4.55) | 115-175 | 11.0/11.2[d] |
| **13** | 337;396 | 1138 | 1285 | 215 (3.97); 289 (4.29) | 125-185 | 9.6/9.8[d] |
| **14** | 340;398 | 1134 | 1283 | 215 (3.96); 289 (4.37) | 105-115 | 4.5/4.6[e,f] |
| **15** | 335;380 | 1138 | 1288 | 215 (3.96); 289 (4.40) | 125-170 | 6.4/6.7[g] |
| **16** | 330;384 | 1138 | 1290 | 215 (3.97); 289 (4.31) | 100-120 | 12.2/12.3[h,i] |
| **17** | 340;399 | 1139 | 1287 | 215 (3.95); 289 (4.62) | 100-110 | 2.8/3.1[j,k] |

[a] FTIR spectra of thin films of pure compound; [b] In ethanol; [c] Weight loss, % (only the first step, together with the corresponding temperature range shown), corresponding to: [d] $4H_2O$ ; [e] $2H_2O$; [f] Another step occurs at 120-180°C, corresponding to $4H_2O$ (9.0/9.3%); [g] 3 $H_2O$; [h] 6 $H_2O$; [i] Another step at 125-170°C, corresponding to $3H_2O$ (6.0/6.1%); [j] 1 $H_2O$; [k] another step at 120-160°C (6.1/6.2%, corresponding to $2H_2O$).

In the IR spectra of complexes **8-17**, the following modifications were evidenced, as compared to the IR spectrum of the ligand **6**, or its sodium salt **7**: (i) important changes in the region 1100-1350 $cm^{-1}$, where the $SO_2$ vibrations appear. Thus, in ligand **6**, the symmetrical vibration appears at 1150 $cm^{-1}$, whereas the asymmetrical one at 1342 $cm^{-1}$. In the sodium salt **7**, the corresponding frequencies are only 2-3 $cm^{-1}$ shifted towards higher wavenumbers, but in all complexes **8-17**, important shifts towards lower wavenumbers occur: with 52-62 $cm^{-1}$ for the asymmetrical vibrations, and with 11-16 $cm^{-1}$ for the symmetrical one. Besides, this last vibration is splitted in the spectra of all complexes **8-17** (data not shown), as an extra band at 1160 $cm^{-1}$ appears. Mention should be made that a similar behavior was documented for other $SO_2$ vibrations in complexes of heterocyclic sulfonamides of types **1-5**, previously reported by this and Borras' groups; [1-10] (ii) the absence of ν(NH) vibrations in the spectra of complexes **8-17** and the sodium salt **7**, whereas in diazoxide **6** they appear at 3080 $cm^{-1}$ ; (iii) the presence of ν(OH) bands, around 3400 $cm^{-1}$,in the spectra of the coordination compounds, which are absent in the spectra of the ligand and its sodium salt(data not shown); (iv) appearance of bands in the region 300-400 $cm^{-1}$ attributed to ν(M-N) and ν(M-O) vibrations, in the spectra of complexes **8-17**, which are again absent in the spectra of **6** and **7**; (v) the other bands in the IR spectra of compounds **8-17** (for instance ν(C=N) and ν (C=C), in the region 1400-1600 $cm^{-1}$ ) appear at the same wavenumbers as in the ligand **6**, probably due to the fact that the part of the molecule in which they are present is not much affected by interaction with the metal ions (data not shown).

In the electronic spectra (in solution) of diazoxide **6**, two absorption maxims are seen, at 215 and 278 nm, respectively, as for other structurally-related benzothiadiazine-1,1-dioxides possessing a similar substitution pattern.[16c,22] For the sodium salt **7**, the first maximum is identical with that of diazoxide **6**, whereas the second one undergoes a bathochromic shift at 289 nm (and a small hyperchromic effect).[22] In the new complexes **8-17**, a similar behavior to that of the sodium salt was evidenced (Table II), as the first maximum remained unchanged, whereas the second one was bathochromically shifted to 289 nm. Such a pattern of the electronic spectra proves that in complexes **8-17** it is the diazoxide anion interacting with the metal ions, similarly with heterocyclic sulfonamides of type **1-5**, which generally coordinate metal ions as deprotonated species, $RSO_2NH^-$.[2-9]

Table III: Diffuse reflectance spectra, magnetic moments and proposed geometries for complexes **8-17**.

| **Complex** | **Electronic spectra** (ν, $cm^{-1}$)$^a$ | $\mu_{eff}$ (BM)$^b$ | **Geometry** |
|---|---|---|---|
| **8** | 25,600; 18,500(sh); 15,630 | 5.28 | octahedral |
| **9** | 17,000; 12,600 | 3.43 | octahedral |
| **10** | 16,200 | 1.88 | distorted octahedral |
| **11** | c | d | tetrahedral |
| **12-14, 16** | c | d | octahedral |
| **15** | 24,600; 20,300; 10,400 | 5.77 | octahedral |
| **17** | 25,900; 15,500; 11,900(sh) | 1.85 | square pyramidal |

[a] In MgO as standard material; [b] At room temperature; [c] No transitions in this spectral region seen;

[d] Diamagnetic.

Reflectance diffuse (RD) spectra of complexes containing colored metal ions are shown in Table III, together with magnetic moment data (at room temperature) and the proposed geometries of the respective metal ions in their complexes with diazoxide. From the above data, it is seen that the Co(II) complex **8** shows two bands in the RD spectrum, at 25,600 and 15,630 $cm^{-1}$ , respectively, assigned as the $\nu_3$ and $\nu_2$ transitions, and a shoulder at 18,500 $cm^{-1}$. The $\nu_1$ calculated from the Lever tables is 7,260 ,which leads to a $\nu_2/\nu_1$ ratio in the range of 2.1-2:2, which correlated with a magnetic moment of 5.28 BM at room temperature, suggests an octahedral geometry for the Co(II) ion.[23] This is also supported by TG analysis data (Table II), which proved that the four water molecules are lost in a single step, between 120-170 °C, being coordinated to the metal ion. For the Ni(II) complex **9**, two weak transitions were evidenced in the RD spectrum, at 17,000 and 16,200 $cm^{-1}$ attributed to the $\nu_1$ and $\nu_2$ transitions of Ni(II) in octahedral surrounding.[5,6] This is also supported by the magnetic moment of 3.43 BM.[6,24] The Cu(II) complex **10**

shows a large structureless band centered at 16,200 $cm^{-1}$ and a magnetic moment of 1.88 BM, indicating probably a distorted octahedral geometry of Cu(II).[6] In the last two complexes water is also directly bound to the metal ions, since by means of TG analysis it was shown that this is lost in one step, between 120-180°C.

The other metal ions showing an interesting RD spectrum in their complexes with diazoxide are Fe(III) and V(IV). Thus, the iron derivative **15** is in its predilect geometry - octahedral - as proved both by spectral as well as magnetic data (Table III), whereas the V(IV) derivative **17** in square pyramidal surrounding, as for the related complexes of sulfonamides **1-5**, containing these metal ions.[4-7,25]

Geometries of other metal ions in the complexes with diazoxide were inferred taking into account the stoichiometry as well as TG data. Thus, the Zn(II) complex is probably in tetrahedral surrounding, as only two water molecules are present in its molecule, which are lost again at high temperature, whereas for Cd(II); Hg(II) and Al(III) complexes, an octahedral geometry of the metal ions is suggested. Some of these complexes also contain lattice water, which was the first lost during the TG analysis (between 100-120°C), followed by the coordinated water molecules, lost at higher temperatures (Table II).

Conductimetry (data not shown) in DMF solution (1 mM), at room temperature, proved complexes **8-17** as well as ligand **6** to be non-electrolytes, whereas the sodium salt **7** was an 1:1 electrolyte.

From the above data it can be concluded that diazoxide **6** is a monodentate ligand, interacting with metal ions by means of the ionized sulfonamide-type nitrogen atom (N-2) of the benzothiadiazine ring. This behavior is very similar to that of saccharin **5**, which is also a monodentate ligand, complexing metal ions by means of the ionized nitrogen atom.[9,10] The prepared complexes are generally octahedral, probably with two diazoxide anions coordinated in trans of each other and four water molecules in the equatorial plane (for the divalent ions, such as Co(II); Ni(II); Cd(II), etc.), or they may possess a different geometry (the Zn(II) or V(IV) derivatives). The complexes of the trivalent metal ions (Al(III) and Fe(III) ) are again octahedral, with three diazoxide anions and three water molecules coordinated.

The prepared complexes were tested for their ability to inhibit carbonic anhydrase isozymes CA I (human) and CA II (bovine), which are the major components of erythrocyte CA (Table IV).

Table IV : CA I and II inhibition data, for $CO_2$ hydration, with compounds **1, 5-17**, determined by Maren's method.[20] For comparison data of a strong (1) and a weak (5) CA inhibitor are also included.

| Compound | $IC_{50}$ (μM)[a] CA I | CA II |
|---|---|---|
| **1** | 0.2[b] | 0.07[b] |
| **5** | 188 | 97[c] |
| **6** | 106 | 54 |
| **8** | 84 | 90 |
| **9** | 91 | 105 |
| **10** | 44 | 40 |
| **11** | 102 | 145 |
| **12** | 86 | 100 |
| **13** | 37 | 35 |
| **14** | 72 | 60 |
| **15** | 91 | 98 |
| **16** | 119 | 160 |
| **17** | 105 | 125 |

[a] Molarity of inhibitor producing a 50% decrease of enzyme specific activity for the $CO_2$ hydration reaction, at 0°C;[b] From refs.[11]; [c] From ref.[10]

As seen from the above data, diazoxide is a much weaker CA inhibitor as compared to acetazolamide 1 (one of the very strong inhibitors), and this is due to the fact that the sulfonamido group is substituted (being of the type $SO_2NH$-X, not $SO_2NH_2$). Generally, it is well documented[1,2] that such a substitution pattern leads to decreased CA inhibitory properties. Still, saccharin, which possesses the same moiety as diazoxide, has CA inhibitory properties in the micromolar range,[10] which is also the case with the last compound. Taking into account that such drugs are used in high enough doses, it might be possible that even such a weak enzyme inhibitory effect might trigger physiological responses. It is interesting to note on the other hand, that the metal complexes prepared in this study are generally weaker inhibitors too, only some of them being more effective than the lgand, a situation diverse from that of the complexes of heterocyclic sulfonamides, which act as very potent CA inhibitors for both isozymes studied here.[1-9] In this context, the strongest inhibitors are the Cu(II) and Hg(II) derivatives **10** and **13**. Probably these compounds are the most effective in inhibiting the proton shuttle of these enzymes, by binding to His-64, as proved for some of their salts by Silverman's group.[26] It is to mention too, the slightly better CA I inhibition with the complexes, as compared to CA II, which is a rare case, since sulfonamides have higher affinities to the last isozyme.

**References**

1. C.T.Supuran,.*Roum.Chem.Quart.Rev.*, **1993**, *1*, 77-116.
2. C.T.Supuran, in "Carbonic Anhydrase and Modulation of Physiologic and Pathologic Processes in the Organism", I.Puscas Ed., Helicon, Timisoara 1994, pp. 29-111.
3. G.Alzuet, S.Ferrer, J.Borras and C.T.Supuran, *Roum.Chem.Quart.Rev.*, **1994**, *2*, 283-300.
4. a) C.Luca, M.Barboiu and C.T.Supuran, *Rev.Roum.Chim.*, **1991**, *36*, 1169-1173; b) C.T.Supuran, *Rev. Roum.Chim.*, **1992**, *37*, 849-855.
5. a) C.T.Supuran, M.Andruh, and I.Puscas, *Rev.Roum.Chim.*, **1990**, *35*, 393-398; b) S.Ferrer, A.Jimenez and J.Borras, *Inorg.Chim.Acta*, **1987**, *129*, 103-106; c) S.Ferrer, G.Alzuet and J.Borras, *J.Inorg.Biochem.*, **1989**, *37*, 163-174; d) C.T.Supuran, G.Manole and I.Manzatu, *Rev.Roum.Chim.*, **1992**, *37*, 739-744.
6. a) C.T.Supuran, G.Manole and M.Andruh, *J.Inorg.Biochem.*, **1993**, *49*, 97-104: b) G.Alzuet, S.Ferrer, J.Borras, A.Castineiras, X.Solans and M.Font-Bardia, *Polyhedron*, **1992**, *11*, 2849-2856; c) C.T.Supuran and M.Andruh, *Rev.Roum.Chim.*, **1994**, *39*, 1229-1234. d) G. Alzuet, J.Casanova, J.Borras, J.A.Ramirez and O.Carugo, *J.Inorg.Biochem.*, **1995**, *57*, 219-234
7. a) M.Andruh, E.Cristurean, R.Stefan, C.T.Supuran, *Rev.Roum.Chim.*, **1991**, *36*, 1727-1732; b) C.T.Supuran, G.Loloiu and G.Manole, *Rev.Roum.Chim.*, **1992**, *37*, 1181-1189; c) C.T.Supuran, R.Olar, D.Marinescu and M.Brezeanu, *Roum.Chem.Quart.Rev.*, **1993**, *1*, 193-204.
8. a) C.T.Supuran, *Metal Based Drugs, in press*; b) L. Sumalan, J.Casanova, G.Alzuet, J.Borras, A.Castineiras and C.T.Supuran, *J.Inorg.Biochem., in press*
9. C.T.Supuran, G.Loloiu and G.Manole, *Rev.Roum.Chim.*, **1993**, *38*, 115-122.
10. C.T.Supuran and M.D.Banciu, *Rev.Roum.Chim.*, **1991**, *36*, 1345-1353.
11. a)T.H.Maren, *Physiol.Rev.*, **1967**, *47*, 595-781; b) T.H.Maren, *Drug Dev.Res.*, **1987**, *10*, 255-276.
12. R.O.Roblin and J.W.Clapp, *J.Am.Chem.Soc.*, **1950**, *72*, 4890-4892.
13. T.H.Maren, *J.Glaucoma*, **1995**, *4*,49-62.
14. I.M.Weiner, in "The Pharmacological Basis of Therapeutics", 8th Edition, A.G.Gilman, T.W.Rall, A.S.Nies and P.Taylor Eds., Pergamon Press, New York, 1990, pp. 708-731.
15. I.Puscas, *Ann.N.Y.Acad.Sci.*, **1984**, *429*, 587-591.
16. a) J.H.Freeman and E.C.Wagner, *J.Org.Chem.*, **1951**, *16*, 815-837; b) F.C.Novello and J.M.Sprague, *J.Am.Chem.Soc.*, **1957**, *79*, 2028-2029; c) F.C.Novello, S.C.Bell, E.L.A.Abrams, C.Ziegler and J.M.Sprague, *J.Org.Chem.*, **1960**, *25*, 970-981; d) J.K.Wales, S.V.Krees, A.M.Grant, J.K.Viktora and F.W.Wolff, *J.Pharmacol.Exp.Ther.*, **1968**, *164*, 421-431.
17. a) M.B.Standen, J.M.Quayle, N.W.Davies, J.E.Brayden, Y.Huang and M.T.Nelson, *Science*, **1989**, *245*, 177-180; b) J.G.Gerben and A.S.Nies, in "The Pharmacological Basis of Therapeutics", 8th Edition, A.G.Gilman, T.W.Rall, A.S.Nies and P.Taylor Eds., Pergamon Press, New York, 1990, pp. 784-813.
18. S.R.Levin, M.A.Charles, M.O'Connor and G.M.Grodsky, *Am.J.Physiol.*,**1975**, *299*, 49-54.
19. C.R.Kahn and Y.Schechter, in "The Pharmacological Basis of Therapeutics", 8th Edition, A.G.Gilman, T.W.Rall, A.S.Nies and P.Taylor Eds., Pergamon Press, New York, 1990, pp. 1463-1495.
20. T.H.Maren, *J.Pharmacol.Exp.Ther.*, **1960**, *130*, 26-29.
21.a) B.Calesnick, B.Katchen and J.Black, *J.Pharm.Sci.*, **1965**, *54*, 1277-1280; b) A.W.Pruitt, B.A.Faraj and P.G.Dayton, *J.Pharmacol.Exp.Ther.*, **1974**, *188*, 248-256.
22. D.V.Parke and R.T.Williams, *J.Chem.Soc.*, **1950**, 1760-1763.
23. L.Banci, A.Bencini, C.Benelli, D.Gatteschi and C.Zanchini, *Struct.Bonding*, **1982**, *52*, 37-79.
24. B.N.Figgis, "Introduction to Ligand Field Theory", J.Wiley &Sons, New York, 1966.
25. C.J.Balhausen and H.Gray, *Inorg.Chem.*, **1962**, *1*, 111-115.
26. C.K.Tu, G.C.Wynns and D.N.Silverman, *J.Biol.Chem.*, **1981**, *256*, 9466-9469.

**Received: November 13, 1995 - Accepted: December 1, 1995 - Received in revised camera-ready format: December 19, 1995**

